# A novel pair of inducible expression vectors for use in *Methylobacterium extorquens*

**DOI:** 10.1186/1756-0500-6-183

**Published:** 2013-05-06

**Authors:** Lon M Chubiz, Jessica Purswani, Sean Michael Carroll, Chistopher J Marx

**Affiliations:** 1 Department of Organismic and Evolutionary Biology, Harvard University, 16 Divinity Ave., Cambridge, MA 02138, USA; 2 Environmental Microbiology Group, Institute of Water Research, University of Granada, C/Ramón y Cajal no. 4, 18071 Granada, Spain; 3 Faculty of Arts and Sciences Center for Systems Biology, Harvard University, Cambridge, MA, USA

## Abstract

**Background:**

Due to the ever increasing use of diverse microbial taxa in basic research and industrial settings, there is a growing need for genetic tools to alter the physiology of these organisms. In particular, there is a dearth of inducible expression systems available for bacteria outside commonly used *γ*-proteobacteria, such as *Escherichia coli* or *Pseudomonas* species. To this end, we have sought to develop a pair of inducible expression vectors for use in the *α*-proteobacterium *Methylobacterium extorquens*, a model methylotroph.

**Findings:**

We found that the P _*R*_ promoter from rhizobial phage 16-3 was active in *M. extorquens* and engineered the promoter to be inducible by either *p*-isopropyl benzoate (cumate) or anhydrotetracycline. These hybrid promoters, P _*R/cmtO*_ and P _*R/tetO*_, were found to have high levels of expression in *M. extorquens* with a regulatory range of 10-fold and 30-fold, respectively. Compared to an existing cumate-inducible (10-fold range), high-level expression system for *M. extorquens*, P _*R/cmtO*_ and P _*R/tetO*_ have 33% of the maximal activity but were able to repress gene expression 3 and 8-fold greater, respectively. Both promoters were observed to exhibit homogeneous, titratable activation dynamics rather than on-off, switch-like behavior. The utility of these promoters was further demonstrated by complementing loss of function of *ftfL* - essential for growth on methanol - where we show P _*R/tetO*_ is capable of not only fully complementing function but also producing a conditional null phenotype. These promoters have been incorporated into a broad-host-range backbone allowing for potential use in a variety of bacterial hosts.

**Conclusions:**

We have developed two novel expression systems for use in *M. extorquens*. The expression range of these vectors should allow for increased ability to explore cellular physiology in *M. extorquens*. Further, the P _*R/tetO*_ promoter is capable of producing conditional null phenotypes, previously unattainable in *M. extorquens*. As both expression systems rely on the use of membrane permeable inducers, we suspect these expression vectors will be useful for ectopic gene expression in numerous proteobacteria.

## Background

As the amount of bacterial genome sequencing information continues to grow, the need for broad-host-range, extensible genetic tools will become increasingly ubiquitous. In particular, the capacity for heterologous gene expression in diverse microbial taxa will be of paramount importance for numerous research goals, as well as industrial and synthetic biological applications. To this end, we explored the use of two well-characterized transcriptional repressors (TetR and CymR) in conjunction with a phage-derived promoter (P _*R*_ from phage 16-3) to produce a novel of set inducible expression vectors for use in the facultative methylotroph *Methylobacterium extorquens*.

Methylotrophic bacteria are a ubiquitous group of microorganisms defined by their capacity to utilize reduced single-carbon (C_1_) compounds as a sole source of energy and biomass. The facultatively methylotrophic, *α*-proteobacterium *Methylobacterium extorquens* has been a model organism in the study of C_1_ metabolism for over 50 years. In the last decade, due in part to the development of a repertoire of genetic tools [1-4]*Methylobacterium* species have become increasingly useful in the study of horizontally transferred metabolic pathways [5-7] and microbial evolution [8-10]. Furthermore, in the past few years genome sequences have become available for eight representatives within *Methylobacterium*[11,12]. While considerable progress has been made for genetic manipulation of *M. extorquens*, an area that remains underrepresented by comparison is the development of regulated expression systems.

To date, only one regulated expression system has been demonstrated to be functional in *M. extorquens*. Choi and coworkers constructed an inducible expression system utilizing the cumate responsive transcriptional repressor, CymR, from *Pseudomonas putida* F1 and the strong P _*mxaF*_ promoter that drives the expression of methanol dehydrogenase in *M. extorquens*[13]. This hybrid system has been modified and utilized to test the fitness consequences of gene expression levels of different formaldehyde oxidation enzymes in *Methylobacterium*[14,15]. While functional, this promoter-operator pairs are extremely “leaky”, wherein the basal level of expression in non-inducing conditions is quite high [14]. This limitation makes heterologous gene expression exceedingly difficult, and hampers the exploration of conditionally null phenotypes.

**Figure 1:**
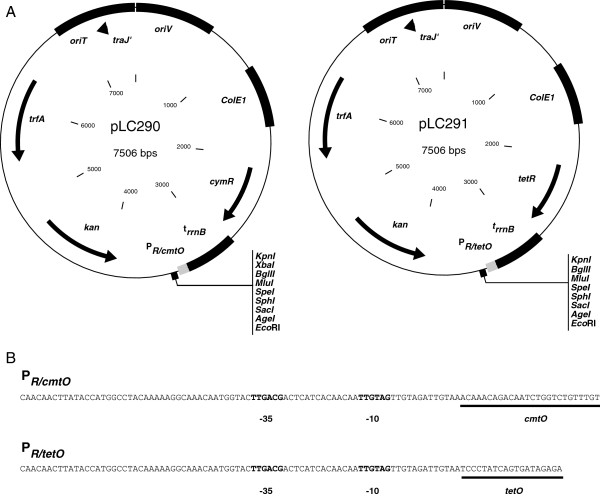
**Map of expression vectors and sequences of P**_***R/cmtO ***_**and P**_***R/tetO***_**.** (**A**) Vectors maps of pLC290 (cumate inducible) and pLC291 (aTc inducible) expression vectors. The multiple cloning site contains a variety of common, single-cutting restriction sites. *oriT*: RP4/RK2 transfer origin. *t*_*rrnB*_: transcriptional terminator. *trfA, oriV*: RK2 (*IncP*) replication protein and origin. *ColE1*: High-copy replication origin for *E. coli* (**B**) Sequences for used for the P _*R/cmtO*_ and P _*R/tetO*_ hybrid promoters. Known RNA polymerase interaction sites (bold text) and engineered operators (underlined) are indicated.

Building on these previous findings, we have employed an additional transcriptional repressor, TetR, from the transposon Tn*10*. As the foundational member of the TetR-family of DNA binding proteins [16], to whom CymR is also a member, TetR has been extensively studied yielding much data on ligand binding, DNA binding kinetics, and operator site specificity [17]. In the absence of inducer, TetR and CymR bind tightly to their respective operator sites (see Figure 1), thereby inhibiting transcriptional initiation by RNA polymerase. Upon binding of ligands such as tetracycline or anhydrotetracycline (a high-affinity ligand) in the case of TetR, or cumate (*p*-isopropyl benzoate) with CymR, the affinity of TetR and CymR for their respective operator sites is nearly abolished, allowing for transcription initiation to proceed. Exploiting these characteristics, numerous studies have modified existing expression systems to behave in a dose-dependent manner. In fact, TetR and related transcriptional repressors have found use in numerous synthetic biology applications in bacteria, archaea, and eukaryotes [13,18-23].

Here we describe the construction of two *IncP*-based, inducible expression vectors for use in *M. extorquens*, and possibly numerous other proteobacteria with minor modification. The novelty of these vectors lies in their use of two separate transcriptional repressors, TetR and CymR, along with a strong promoter from the rhizobial phage 16-3. We demonstrate the utility of these vectors by showing that i) induction is dose-dependent, ii) induction is continuous through time, and iii) the regulatory range of both systems exceeds those currently available for *M. extorquens*. Collectively, these results supply researchers investigating *M. extorquens*, and likely numerous other proteobacteria, with two alternative systems to express genes in traditional and synthetic biology applications.

## Findings

### Promoter design and rationale

During the process of selecting an appropriate promoter, we desired that the promoter i) be sufficiently active in *M. extorquens* and ii) not be subject to regulation by native transcription factors. Based on these two criteria, a natural source for such a promoter was from bacteriophage. Many bacteriophage promoters have a wide host range and often have strong, constitutive activity in the absence of their transcriptional control mechanisms. However, numerous well characterized coliphage-derived promoters such as *λ* P _*L*_, *λ* P _*R*_, T5 P _*N*25_, T7 P _*A*1_ are weakly active or inactive in *M. extorquens*[13]. To this end, we looked to other bacteriophage promoters that have been shown to be active in *α*-proteobacteria. Based on this metric, we explored the use of promoters from the control region of the rhizobial phage 16-3 (P _*L*_ and P _*R*_). Phage 16-3 has been extensively examined with physiological and biochemical studies in both its host, the *α*-proteobacterium *Sinorhizobium meliloti*, and *Escherichia coli*[24,25], suggesting that P _*L*_ and P _*R*_ may be functional in a variety of hosts. Additionally, the only transcriptional regulator known to interact with P _*L*_ and P _*R*_ is the 16-3 C repressor [25].

**Figure 2:**
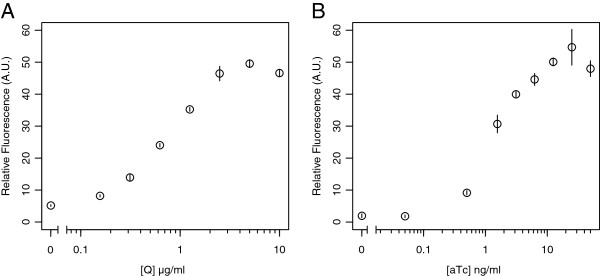
**Induction profiles of the P**_***R/cmtO ***_**and P**_***R/tetO ***_**promoters.** Induction profiles of *mCherry* containing pLC290 and pLC291 derivatives (**A**) pJP18T and (**B**) pJP22T in *M. extorquens* PA1. Cell cultures were grown to mid-log phase and induced for 24 hrs prior to fluorescence measurements. Fluorescence units are presented as arbitrary units (A.U.) and normalized as described in Materials and Methods.

In a set of exploratory experiments, we found that P _*R*_ was active in *M. extorquens* (data not shown). As we desired to construct inducible systems, we focused attention to engineering P _*R*_ derivatives containing operator sites for the CymR and TetR regulators (Figure 1). The resulting hybrid promoters, P _*R/cmtO*_ and P _*R/tetO*_, were found to produce the widest regulatory range without interfering with P _*R*_ promoter activity. Interestingly, we found that placing the operators, specifically *tetO*, throughout other regions of the promoter resulted in either loss of promoter repression or activity (data not shown). This was a somewhat surprising result given the flexibility of many other phage-derived systems to be manipulated with multiple repressor and activator operator sites [18,26]. Collectively, these findings allowed us to engineer two inducible promoters with similar maximal activity (Figure 2).

### Activation of P _*R/cmtO*_ and P _*R/tetO*_ is dose-dependent

A desirable property for regulated expression systems is for levels of gene expression from the promoter to be proportional to the concentration of inducer. In order to explore the range of induction of P _*R/cmtO*_ and P _*R/tetO*_, the promoters along with their respective regulatory proteins were introduced onto broad-host-range plasmids (*IncP* compatibility group) to create the expression vectors pLC290 and pLC291 (Figure 1). Since previous studies have demonstrated *mCherry* to be a sensitive measure of gene expression in *M. extorquens*[14], we decided to use mCherry fluorescence as a metric of promoter activity. We placed the red-fluorescent protein variant *mCherry* under the control of each promoter in pLC290 and pLC291 and introduced the resulting vectors (pJP18T and pJP22T) into *M. extorquens*. To induce expression from P _*R/cmtO*_ and P _*R/tetO*_, we supplied varied concentrations of cumate (Q) and anhydrotetracycline (aTc), respectively, to *M. extorquens* cultures.

In general, both promoters were found to be responsive to concentrations of Q and aTc that were in agreement with previous studies in *M. extorquens* or other organisms [13,18,27]. The P _*R/cmtO*_ promoter was observed to respond to a range of 0.1 to 5 µg/ml (0.6 to 30 µM) of Q and the P _*R/tetO*_ promoter from 0.1 to 25 ng/ml (0.2 nM to 50 nM) aTc. Interestingly, the induction profile of P _*R/cmtO*_ increased in a log-linear fashion over the entire concentration range, whereas P _*R/tetO*_ was observed to have a much more concave profile. In terms of regulatory range, P _*R/cmtO*_ and P _*R/tetO*_ were observed to have 10-fold and 30-fold induction, respectively, with both promoters having the same maximum absolute levels of expression (Figure 2). Importantly, the basal level of expression from P _*R/cmtO*_ was found to be approximately 3-fold higher than that of P _*R/tetO*_. Taken together, these data suggest that while P _*R/cmtO*_ may be more tunable, P _*R/tetO*_ serves as a superior expression system for genes requiring tight repression, such as cytotoxic proteins. Also, we found that there was minimal cross-talk between the CymR and TetR ligand specificity or promoter binding indicating these systems would work independent of one another (pJP18T: 4.6 Uninduced/4.2 with aTc; pJP22T: 1.0 Uninduced/1.1 with Q; Grown in succinate).

Comparing the levels of gene expression and regulatory range of P _*R/cmtO*_ and P _*R/tetO*_ to the cumate inducible P _*mxaF*_ promoter previously reported [13,14], we found that in *M. extorquens* these promoters achieve 33% of the maximal activity of P _*mxaF*_ (the strongest known *Methylobacterium* promoter) and provide a greater degree of repression. Specifically, a cumate-inducible P _*mxaF*_*mCherry* expression vector, pHC115m, yielded relative fluorescence values of 15.6 ± 1.5 (uninduced) to 157.1 ± 3.7 (induced). While this 10-fold regulatory range was similar to P _*R/cmtO*_, the minimal and maximal expression from P _*R/cmtO*_ were both 3-fold lower. By comparison, P _*R/tetO*_, with a 30-fold regulatory range, was able to repress expression 8-fold lower than the P _*mxaF*_ system with only a 3-fold difference in maximum expression. Collectively, these results demonstrate that both P _*R/cmtO*_ and P _*R/tetO*_ provide improvement over previously explored systems. However, we do note that P _*mxaF*_ may remain a superior promoter in cases when high-level protein over-expression is desired. Importantly, these hybrid promoters allow for more relevant exploration of cellular physiology as their expression levels and ranges fall well within or above native promoters in *M. extorquens*.

### Maximal activation of P _*R/cmtO*_ and P _*R/tetO*_ is substrate dependent

An issue with many expression systems designed with host-derived promoters is the possibility of interactions with native transcription factors. Specifically, the P _*mxaF*_ promoter is known to be more highly active in cells grown on methanol as opposed to succinate [1,28]. To explore this possibility, with respect to P _*R/cmtO*_ and P _*R/tetO*_, we cultured *M. extorquens* harboring pJP18T and pJP22T in media with either methanol or succinate as the sole carbon source (Table 1). We found that succinate grown cells possessed a nearly 2-fold increase in maximal gene expression, compared to methanol grown cells; effectively, the opposite behavior seen with P _*mxaF*_. We suspect that this disparity in maximal expression may be due to an external factor, such as different plasmid copy numbers, between methanol and succinate growth. Previously reported XylE and *β*-galactosidase promoter probe vectors used in *M. extorquens*, such as pCM130 and pCM132 (plasmids with the same backbone as pLC290 and pLC291), exhibit between 2 and 3-fold increases in background activity during succinate versus methanol growth [1]. As pCM130 and pCM132 possess no promoter sequences upstream of their reporter genes, the only likely variation that might exist is in plasmid copy number. Comparing these findings to our own, where P _*R/cmtO*_ and P _*R/tetO*_ contain no host-related transcription factor binding sites, we see similar fold changes in maximal expression suggesting that a similar mechanism may be affecting these expression systems. Taken together, these data indicate that single-copy or chromosomally integrated systems be used in situations where uniform expression is desired across substrates.

**Table 1:** **Growth substrate dependence on P**_***R/cmtO ***_** and P**_***R/tetO ***_**activation**

		**Methanol**		**Succinate**	
**Plasmid**	**Promoter**	**Uninduced**	**Induced**	**Uninduced**	**Induced**
pJP18T	P _*R/cmtO*_	5.32 ± 0.64	28.94 ± 2.14	5.79 ± 0.44	61.93 ± 2.42
pJP22T	P _*R/tetO*_	1.46 ± 0.27	33.13 ± 2.63	1.95 ± 0.62	54.67 ± 5.60

Gene expression as measured by *mCherry* fluorescence from *M. extorquens* cells harboring pJP18T or pJP22T. Cells were grown in succinate or methanol medium in the presence or absence of Q (5 µg/ml) or aTc (25 ng/ml). Values are relative fluorescence (arbitrary units) and reported error is the 95% confidence interval (N = 4).

### Induction of P _*R/cmtO*_ and P _*R/tetO*_ is continuous

**Figure 3:**
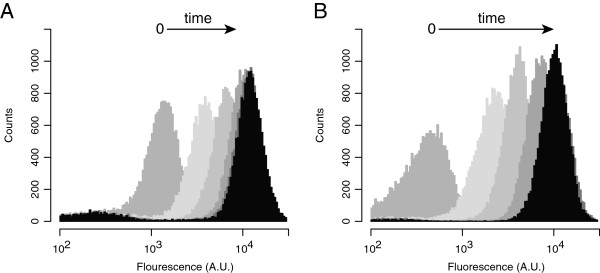
**Single-cell dynamics of P **_**R/cmtO **_**and P**_**R/tetO **_**activation.** Histograms of relative fluorescence values for pJP18T (**A**) and pJP22T (**B**) harboring *M. extorquens* PA1 as determined by single-cell flow cytometry. Cultures were grown to mid-log phase and induced with 5 µg/ml Q (**A**) or 25 ng/ml aTc (**B**). At times 0, 2, 4, 6, 8, and 24 hrs, cells were harvested and fixed in carbon-free Hypho medium supplemented with 100 mg/ml streptomycin. The 8 and 24 hr time points have nearly overlapping fluorescence distributions. Fluorescence units are presented as arbitrary units (A.U.) and normalized as described in Materials and Methods.

A problematic feature of many expression systems, particularly those associated with metabolic pathways, is that gene expression can exhibit phenotypic heterogeneity throughout the population of cells, such as an on-off, switch-like behavior [29-31]. To explore this possibility, we grew *M. extorquens* strains bearing the *mCherry* expression vectors pJP18T and pJP22T to mid-log phase, induced cultures with either Q or aTc, and measured the time course of individual-cell fluorescence by flow cytometry. We found that over 8 hours of induction the induced populations activated transcription in a uniform, continuous manner (Figure 3). Though we did observe residual uninduced cells, we suspect this may be due to debris introduced by our cell fixing method or possibly cells losing *mCherry* due to costly over-expression. These data demonstrate the utility of the P _*R/cmtO*_ and P _*R/tetO*_ expression systems in studying aspects of cellular physiology requiring uniform gene expression.

### Complementation and conditional null phenotypes using P _*R/tetO*_ constructs

**Figure 4:**
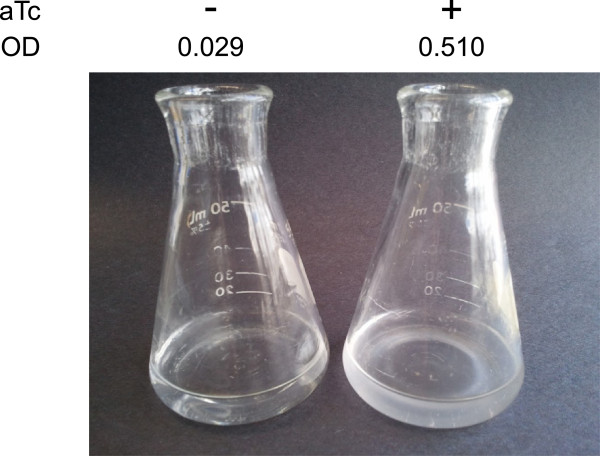
**Complementation and conditional null phenotype of *ftfL*.** Images of methanol grown cultures of *M. extorquens* AM1 strains CM4103 in the presence (+) and absence (-) of 20 µg/ml aTc. Final OD(600 nm) values presented are after 72 hrs of growth at 30°C in 20 mM MeOH supplemented medium

To examine the utility of these vectors for studying *M. extorquens* physiology, we complemented a gene encoding a key enzyme in methanol metabolism using the P_*R/tetO*_-based plasmid pLC291. We chose to use utilize P _*R/tetO*_ due to the tight induction properties we have observed using an mCherry reporter (Figure 2 and Table 1). The product of *ftfL* (formate-tetrahydrofolate ligase) is required for the assimilation of formate into biomass during one-carbon metabolism [32]. A disruption in *ftfL* results in a methanol minus growth phenotype. By complementing a *ftfL* knockouts using *ftfL*–expressing vectors under the control of P _*R/tetO*_, in the presence of aTc, we found that we could fully restore growth on methanol (Figure 4). Importantly, in the absence of aTc, we observed that we were able to produce a complete null phenotype for *ftfL* (Figure 4). To date, no expression system for *M. extorquens* has been capable of producing conditional null phenotypes. These results demonstrate the utility of P _*R/tetO*_ to study M. extorquens physiology and generate conditional null mutants regulated by aTc.

## Conclusions

To date, only a handful of expression systems exist for bacterial models outside *E. coli* and other closely related *γ*-proteobacteria. In an effort to expand the genetic toolkit available to researchers working with *M. extorquens*, and presumably other proteobacteria, we have constructed a set of two inducible expression vectors that utilize the CymR and TetR (cumate and tetracycline repressors) in conjunction with the strong P _*R*_ promoter from phage 16-3. The pLC290 and pLC291 vectors were found to provide uniform, high-level expression in *M. extorquens* over a wide range of inducer concentrations. Importantly, compared to the only existing inducible system for *M. extorquens*, we found that P _*R/cmtO*_ and P _*R/tetO*_ have 3 and 8-fold increases in repression, respectively. This provides a significant improvement in the ability to explore *M. extorquens* cellular physiology. Further, as these promoters operate orthogonally to one another, we believe these expression systems will easily work in concert within a single strain to allow complex genetic engineering in a wider range of bacteria. For these reasons, we believe these vectors and promoter systems will be of great use to the bacteriological community in many research and industrial settings.

## Availability of supporting information

The plasmid data supporting the results of this article are available in the AddGene repository with identification numbers http://www.addgene.org/44447/ and http://www.addgene.org/44448/.

## Methods

### Bacterial strains, medium, and growth conditions

All bacterial strains used in this work are derivatives of *Escherichia coli* NEB10 *β* (New England Biolabs), *E. coli* LC100 (F^−^*rph*-1 *ilvG attλ*::[*spcRlacI*^*Q*^*tetR*]) [33], *Methylobacterium extorquens* PA1 strain CM2730 (*ΔcelABCD*) [34] or *M. extorquens* AM1. Growth of all strains, except *E. coli*, was performed in modified ’Hypho’ minimal medium as described by Chou and coworkers [10], with succinate at 5 mM or methanol at 20 mM. *E. coli* strains were cultured in Luria-Bertani broth as described by Miller [35] or nutrient broth. Media was supplemented with kanamycin at 50 µg/ml or ampicillin at 100 µg/ml to select for the presence of all plasmids. Inducers anhydrotetracycline (aTc) and cumate-KOH (Q) were supplied at 25 ng/ml or 5 µg/ml from aqueous stocks, respectively, unless otherwise indicated. Growth and gene expression experiments were performed at 30°C using an automated growth system described by Delaney and coworkers [34,36].

### Plasmid and strain construction

Promoter designs were initially constructed and subsequently mutated in a pBluescript(SK-) (Stratagene) backbone. Synthetic oligonucleotides CAACAACTTATACC ATGGCCTACAAAAAGGCAAACAATGGTACTTGAC GACTCATCACAA and GTCCGTTCGTTACAATCTA CAACTACAATTGTTGTGATGAGTCGTCAAGTACC ATTG containing the sequence for a 91 nt region encoding the P _*R*_ promoter from the rhizobial phage 16-3. The oligonucleotides were annealed to form a 91 bp dsDNA fragment, followed by PCR amplification with primers ATAGGGCCCCAACAACTTATACCATGGCC TAC and ATAGGTACCGTCCGTTCGTTACAATCTA CAAC to introduce *Psp*OMI and *Kpn*I restriction sites. The resulting fragment was digested with *Psp*OMI and *Kpn*I and cloned into the respective sites in pBluescript(SK-) to form pLC265. TetR and CymR operator sites (*tetO* and *cmtO*), were introduced at the distal end of P _*R*_ in pLC265 using enzymatic inverse PCR (EI-PCR) [37] using primers ATACGTCTCATCCCTATCAGTGA TAGAGAGTTGTAGATTGTAACGAACGGAC, ATAC GTCTCAGGGACGTCAAGTACCATTGTTTGCC, AT ACGTCTCAACAAACAGACAATCTGGTCTGTTTGT GGTACCCAATTCGCCCTATAG, and ATACGTCTCA TTGTTTACAATCTACAACTACAATTGTTGTG fol- lowed by *Bsm*BI digestion and ligation to generate plasmids pLC271 (P _*R/tetO*_ containing) and pLC277 (P _*R/cmtO*_ containing).

The subsequent broad-host-range vectors were constructed using the expression vector pHC115 [14] as a template. A DNA region encoding Tn*10tetR* was PCR amplified from LC100 using primers ATAGCT AGCAGGGAGAGACCCCGAATGATGTCTAGATTAG ATAAAAGTAAAGTG and ATAGGGCCCTTAAGACC CACTTTCACATTTAAG containing *Nhe*I and *Psp*OMI restriction sites. The resulting product was digested and ligated into the *Nhe*I and *Psp*OMI sites of pHC115, thereby replacing the *cymR* coding region with *tetR* to form pLC261. From pHC115 and pLC261, the P _*mxaF*_ region was excised with *Psp*OMI and *Kpn*I and replaced with subcloned P _*R/cmtO*_ and P _*R/tetO*_ fragments from pLC277 and pLC271. To the resulting plasmids, a t _*rrnB*_ terminator was PCR amplified from pHC01 [10] using primers ACGCGAAATTCAAGCGC TAGGGCCAAGTTGGGTAACGCCAGGGTTTTCCC or ATGTGAAAGTGGGTCTTAAGGGCCAAGTTGG GTAACGCCAGGGTTTTCCC and TGTAGGCCAT GGTATAAGTTGTTGGGATGCAAAAACGAGGCTAG TTTACC and cloned into the *Psp*OMI site, using the method of Gibson and coworkers [38], to reduce transcriptional read-through into the P _*R/cmtO*_ and P _*R/tetO*_ promoter regions. Likewise a more comprehensive multiple cloning site was introduced into the *Kpn*I and *Eco*RI sites using annealed synthetic oligonucleotides GATAG GTACCTCTAGAAGATCTACGCGTACTAGTGCATG CGAGCTCACCGGTGAATTCATAG and CTATGAAT TCACCGGTGAGCTCGCATGCACTAGTACGCGTAG ATCTTCTAGAGGTACCTATC to produce the final expression vectors pLC290 and pLC291. The *mCherry* expression vectors pJP18T and pJP22T were created by subcloning a *Kpn*I and *Eco*RI digestion product containing *mCherry* from pHC115m [14] into the corresponding sites in pLC290 and pLC291, respectively. The vectors pLC290 (GenBank Accession KC296704) and pLC291 (GenBank Accession KC296705) are publically available from the non-profit organization AddGene.org (http://www.addgene.org/Christopher_Marx).

Unmarked *ftfL* knockouts were generated by transforming the the Cre-recombinase expression plasmid pCM157 [3] into *M. extorquens* AM1 derivatives CM216K.1 [39] generating strain CM2336 (*ΔftfL*::*loxP*). The *ftfL* omplementation vector was generated by subcloning a *Kpn*I and *Eco*RI digestion product of a pHC115-based *ftfL* plasmid (SMC unpublished) into the corresponding sites of pLC291, creating plasmids pSC54. The vector, pSC54, was introduced into CM2336 via triparental mating using the helper plasmid pRK2073 [40,41], to produce strains CM4103 (*ΔftfL*::*loxP*/pSC54). Complementation was performed by inoculation of succinate grown CM4103 into methanol minimal medium containing 0 µg/ml or 20 µg/ml aTc.

### Fluorescence-based expression assays

Assays to measure levels of *mCherry* protein expression were performed as follows. For dose-dependent response curves, *M. extorquens* strains harboring pJP18T or pJP22T were grown to saturation in 10 ml of Hypho-succinate medium. These cultures were then diluted 1:200 in fresh medium, followed by 630 µl aliquots being dispensed to clear, flat-bottom, 48-well microtiter plates (Costar). Cultures were grown for 4 hrs on a plate shaking tower (Caliper) at 150 rpm in a 30°C humidified room. After 4 hrs of growth, 10 µl of fresh medium containing Q or aTc was added to supply Q and aTc at desired concentrations. Cultures were allowed to continue growth for an additional 24 hrs prior to fluorescence (excitation 587 nm/emission 610 nm) and optical density (600 nm) measurements made using a Tecan Safire2 plate reader. Relative fluorescence values reported are:

$$\begin{array}{ll} \text{Relative fluorescence (A.U.)}=\frac{\text{RFU}}{OD_{600}}*10^{-3} & \; \end{array}$$

Dynamic expression assays were conducted under similar conditions as above with the following exceptions. Cells (200 µl of culture) were harvested after induction at 0, 2, 4, 6, 8, and 24 hrs. Culture samples were pelleted by centrifugation (6,000 x*g*) and resuspended in an equal volume of cold Hypho medium without succinate and supplemented with 100 mg/ml streptomycin to inhibit *mCherry* translation. Fixed cells were kept on ice prior to fluorescence measurements made using a BD LSR II Flow Cytometer. Flow cytometry data were then analyzed using the BioConductor *flowCore* package in R [42]. Reported fluorescence values for flow cytometry are raw values from the BD LSR II and were not correlated to those of the Tecan Safire2.

## Competing interests

The authors (LMC, JP, SMC and CJM) declare no competing interests with respect to the findings in this article.

## Authors’ contributions

LMC and CJM were responsible for the conception and design of the study. LMC, JP, and SMC constructed all vectors and conducted all growth and fluorescence measurement experiments. LMC, SMC, and CJM drafted the manuscript. All authors read and approved the final manuscript.
